# Effect of head pitch and roll orientations on magnetically induced vertigo

**DOI:** 10.1113/JP271513

**Published:** 2015-12-30

**Authors:** Omar S. Mian, Yan Li, Andre Antunes, Paul M. Glover, Brian L. Day

**Affiliations:** ^1^ Sobell Department of Motor Neuroscience and Movement Disorders, Institute of NeurologyUniversity College LondonLondonUK; ^2^ Sir Peter Mansfield Imaging CentreUniversity of NottinghamNottinghamUK

## Abstract

**Key points:**

- Lying supine in a strong magnetic field, such as in magnetic resonance imaging scanners, can induce a perception of whole‐body rotation.
- The leading hypothesis to explain this invokes a Lorentz force mechanism acting on vestibular endolymph that acts to stimulate semicircular canals.
- The hypothesis predicts that the perception of whole‐body rotation will depend on head orientation in the field.
- Results showed that the direction and magnitude of apparent whole‐body rotation while stationary in a 7 T magnetic field is influenced by head orientation.
- The data are compatible with the Lorentz force hypothesis of magnetic vestibular stimulation and furthermore demonstrate the operation of a spatial transformation process from head‐referenced vestibular signals to Earth‐referenced body motion.

**Abstract:**

High strength static magnetic fields are known to induce vertigo, believed to be via stimulation of the vestibular system. The leading hypothesis (Lorentz forces) predicts that the induced vertigo should depend on the orientation of the magnetic field relative to the head. In this study we examined the effect of static head pitch (−80 to +40 deg; 12 participants) and roll (−40 to +40 deg; 11 participants) on qualitative and quantitative aspects of vertigo experienced in the dark by healthy humans when exposed to the static uniform magnetic field inside a 7 T MRI scanner. Three participants were additionally examined at 180 deg pitch and roll orientations. The effect of roll orientation on horizontal and vertical nystagmus was also measured and was found to affect only the vertical component. Vertigo was most discomforting when head pitch was around 60 deg extension and was mildest when it was around 20 deg flexion. Quantitative analysis of vertigo focused on the induced perception of horizontal‐plane rotation reported online with the aid of hand‐held switches. Head orientation had effects on both the magnitude and the direction of this perceived rotation. The data suggest sinusoidal relationships between head orientation and perception with spatial periods of 180 deg for pitch and 360 deg for roll, which we explain is consistent with the Lorentz force hypothesis. The effects of head pitch on vertigo and previously reported nystagmus are consistent with both effects being driven by a common vestibular signal. To explain all the observed effects, this common signal requires contributions from multiple semicircular canals.

## Key points

- Lying supine in a strong magnetic field, such as in magnetic resonance imaging scanners, can induce a perception of whole‐body rotation.
- The leading hypothesis to explain this invokes a Lorentz force mechanism acting on vestibular endolymph that acts to stimulate semicircular canals.
- The hypothesis predicts that the perception of whole‐body rotation will depend on head orientation in the field.
- Results showed that the direction and magnitude of apparent whole‐body rotation while stationary in a 7 T magnetic field is influenced by head orientation.
- The data are compatible with the Lorentz force hypothesis of magnetic vestibular stimulation and furthermore demonstrate the operation of a spatial transformation process from head‐referenced vestibular signals to Earth‐referenced body motion.

**Abbreviations**
*B*_0_: The constant, static magnetic field of the MRI scanner
*B*_0_*x*/*B*_0_*y*/*B*_0_*z*: *X*, *Y* and *Z* components of *B*_0_ in the lab coordinate system
d*B*_0_/d*t*: change in static magnetic field with respect to time (due to movement through magnetic field gradient)
MRI: magnetic resonance imaging
SPV: slow phase velocity
*X*_Head_/*Y*_Head_/*Z*_Head_: *X*, *Y* and *Z* axes of the head coordinate system
*X*_Lab_/*Y*_Lab_/*Z*_Lab_: *X*, *Y* and *Z* axes of the lab coordinate system

## Introduction

It is well established that vertigo is sometimes experienced inside magnetic resonance imaging (MRI) scanners (Schenck *et al*. 1992; Glover *et al*. 2007; Theysohn *et al*. 2008; Versluis *et al*. 2013). Most studies have provided only casual characterisation of this phenomenon. However, we recently performed a detailed examination of vertiginous perceptions experienced by supine healthy adults as they were pushed into and then remained in the static uniform field at the centre of a 7 T MRI scanner in the absence of vision, and with other veridical motion cues minimised (Mian *et al*. 2013). Under these conditions, almost all participants experienced vertigo, with the dominant perception being that of rotation in the horizontal plane (i.e. as if the bed is spinning about a vertical axis). On average, the perception of rotation lasted about a minute whilst inside the magnetic field, and then re‐emerged in the opposite direction upon withdrawal from the field.

Several mechanisms have been proposed to account for magnetically induced vertigo via stimulation of the vestibular system. A number of these depend on movement or varying components of the magnetic field, such as its rate of change with respect to time and space (Glover *et al*. 2007). These states are experienced transiently when entering and exiting the magnet, but not whilst stationary at the centre of the magnet. Roberts *et al*. (2011) proposed an alternative mechanism for magnetic vestibular stimulation that does not depend on movement or varying components of the magnetic field. They suggested that the magnetic field interacts with permanent ionic currents, which occur spontaneously in the labyrinthine endolymph, to induce orthogonal Lorentz forces that can push on a semicircular canal cupula if it is orientated appropriately with respect to the magnetic field. With this mechanism, the cupula will experience a constant force throughout exposure to a uniform magnetic field. Even though the vertiginous perception of rotation is temporary under such an exposure, we concluded the Lorentz force hypothesis was the most likely explanation for the dominant vertiginous perception (Mian *et al*., 2013, 2015). This interpretation supposes that the temporary nature of the perception of rotation and its re‐emergence upon withdrawal from the field are due to central adaptation to continuous vestibular input, compatible with prior observations of the non‐continuous perceptual response to other forms of continuous stimulation of the semicircular canals (Guedry & Lauver, 1961; Clark & Stewart, 1968; St George *et al*. 2011). Furthermore, movement and varying components of the field do not appear to be critical factors for other manifestations of magnetic vestibular stimulation, such as nystagmus in humans (Roberts *et al*. 2011) and locomotor circling in rodents (Houpt *et al*. 2007, 2011).

An important aspect of the mechanism is that the direction and magnitude of the induced Lorentz force, and thus activation pattern of the various semicircular canals, is dependent on the orientation of the magnetic field relative to the vestibular system (Roberts *et al*. 2011; Antunes *et al*. 2012). In line with this, the magnitude and direction of nystagmus in humans (Roberts *et al*. 2011) and locomotor circling in rodents (Houpt *et al*. 2013) is modulated by orientation of the head in the magnetic field. In the current study, we examine the influence of head pitch (rotation about head medio‐lateral axis) and head roll (rotation about head antero‐posterior axis) orientations on the vertigo produced when exposed to a strong magnetic field. These manipulations alter the spatial relationship between the magnetic field and vestibular system and thus an absence of influence on vertigo would be incompatible with a Lorentz force mechanism being responsible. We focus on the self‐motion perception of horizontal‐plane rotation as we previously found this to be the dominant component of vertigo, and was amenable to quantification (Mian *et al*. 2013). In the Discussion we will consider whether modulation of this perception by head orientation is compatible with existing observations of the effect of head pitch on magnetically induced nystagmus (Roberts *et al*. 2011). On a practical level, the data reveal which head orientations provide the weakest and which provide the strongest sensations of vertigo‐related discomfort to people exposed to high field MRI.

## Methods

### Ethical approval

The study conformed to the Declaration of Helsinki and received approval from the University of Nottingham Medical School Research Ethics Committee. All participants provided signed, informed consent.

### Participants

All participants were screened by questionnaire to exclude those with general contraindications for the MRI environment, as well as those with neurological or otological conditions and those who suffer from claustrophobia. Eighteen participants were recruited (50% male; age range 18–60 years, mean ± SD = 29 ± 10 years). Twelve took part in experiment 1 (effect of head pitch) and 11 took part in experiment 2 (effect of head roll). Five were common to both. Some neutral head position (0 deg) trials from the current experiments were common with some trials used in Glover *et al*. (2014) and in experiment 2 of Mian *et al*. (2013).

### Coordinate systems

**Figure 1:**
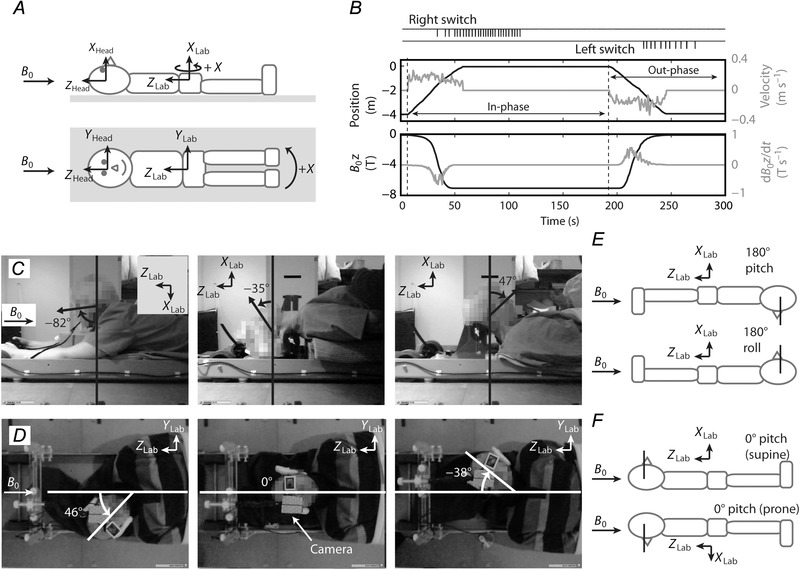
**Experimental set‐up** *A*, cartoon of a participant laying on scanner bed in neutral head position viewed from the side and from above depicting orientation of head and lab coordinate systems used in this paper. Perception of positive rotation about *X*_Lab_ is depicted by the curved arrows. The magnetic field is in the −*Z*_Lab_ direction. *B*, example of bed movement and switch press data from a 0 deg head angle trial (adapted from Mian *et al*. 2013). Middle panel plots head position (black, left axis, 0 m = bore centre) and velocity (grey, right axis) along *Z*_Lab_. Bottom panel shows magnetic field (black, left axis) and its temporal rate of change (grey, right axis) experienced by the head in *Z*_Lab_. Top panel shows voltage output from switches for logging perceived rotation (pulses depict depression of switches). We refer to the portion of the trial between the start of movement into the scanner and start of movement out of the scanner as the in‐phase. Everything after the in‐phase is the out‐phase. *C*, photographs of a participant (before start of trial) at −80, −40 and +40 deg head pitch conditions (+, forward pitch). Annotations indicate actual measured head pitch angles. The −80 and −60 deg conditions were performed prone, whilst other conditions were performed supine. *D*, photographs of a participant at +40, 0 and −40 deg head roll (+, right‐ear‐to‐shoulder). Eye‐wear and camera above eye were used for recording eye movements. *E*, 180 deg pitch and 180 deg roll conditions. *F*, rotating the subject 180 deg about *Z*_Lab_, so they are prone instead of supine, has no impact on head relative to magnetic field. For the trials in this body orientation (–60 and −80 deg head pitch), we report perceptions as if these trials were undertaken supine by also rotating the lab reference system about *Z*_Lab_.

Figure 1 *A* is a cartoon depicting a participant lying supine on the scanner bed for head first entry into the scanner, together with lab and head right‐hand coordinate systems. The *XY*_Head_ plane is Reid's plane (the plane formed by the external auditory canals and the lower orbital margins). The *YZ*_Lab_ plane is earth horizontal. At 0 deg head pitch and 0 deg head roll, the orientations of the head and lab coordinate systems are aligned. All rotations (head angles, perceived rotations, and eye movements) are reported according to the right‐hand grip rule: point thumb in direction of positive axis and fingers curl in direction of positive rotation. For example, forward head pitch orientation (chin‐to‐chest) is a positive rotation about *Y*. Right‐ear‐to‐shoulder head roll is a positive rotation about *X*. For the purpose of describing head translation within the magnetic field (‘magnetic field exposure’ section), the origin of the lab coordinate system is the magnet isocentre.

Perceptions of rotation are described as rotations about the lab coordinate system (although the centre of rotation is considered to be somewhere within the confines of the body rather than magnet isocentre – see Results). Thus, a perception of horizontal‐plane rotation is described as rotation about *X*_Lab_. For the condition in Fig. 1 *A*, the perception of legs rotating to the left would be described as +*X*_Lab_ rotation. This was true for the other conditions with the exception of the 180 deg head pitch condition, where it would be described as −*X*_Lab_ rotation (Fig. 1 *E*). For the −60 and −80 deg head pitch conditions, it was appropriate to rotate the lab coordinate system 180 deg about *Z*_Lab_ (Fig. 1 *F*; see ‘Head orientations’ section for details and justification). Eye movements are described as ocular rotations about the head coordinate system. Thus, a leftward pupil motion is described as a positive ocular rotation about *Z*_Head_ and a pupil motion in the forehead to chin direction is described as a positive rotation about *Y*_Head_. For convenience, we will also use conventional nomenclature for eye movements by referring to ocular rotations about *Z*_Head_ and *Y*_Head_ as horizontal and vertical, respectively, even though at the neutral head position of the current experiments, these eye movements are orthogonal to earth‐horizontal and earth‐vertical planes.

### Procedure

The experiments involved slowly pushing participants into a 7 T Philips Achieva MRI scanner (with Magnex un‐shielded 7T/900 magnet) and logging their perceptions whilst they laid still on a bed with their head in various orientations (see ‘Head orientations’ section for details). No imaging was performed, so only the static magnetic field (*B*_0_) was present, which is uniform over approximately 60 cm at the centre. A field plot of the magnet can be seen in figure 3 of Glover *et al*. (2007). Head orientation had a minor effect on magnetic field exposure during movement into and out of the scanner but not at the centre of the scanner (see ‘Magnetic field exposure’ section for details). A custom‐made track and bed was installed in place of the standard Philips bed (photograph in Mian *et al*. 2013). This was deemed important for two reasons: (1) smooth castors and tracks were used to minimise jerkiness of bed motion into and out of the bore – this, together with exclusion of vision, served to minimise veridical motion information during the experiments; and (2) the frame was constructed to the maximum length possible in the scanner room – this meant that the baseline field exposure of the head at the start of each trial could be much lower (approx. 0.1 T) than could be achieved using the standard scanner bed (approx. 1 T).

For each participant, one trial was performed per head orientation. Prior to the start of each trial, the head was guided to the desired orientation. Padded frames and wedges were used to support the head in the desired position. Vision was then removed (eyes closed or room made dark) and participants remained stationary on the bed until the end of the trial. The bed was manually slowly pushed into the magnet by an experimenter with the aim of covering the distance from the start to the centre of the magnet (approx. 4 m) in approximately 50–60 s. A wall mounted stopwatch was used to aid the experimenter in this task. Once the bed was stationary, the participant was kept at the centre of the magnet for 135 s and then returned to the start position at approximately the same rate as the rate of entry. Pilot tests had indicated that a dwell time of 135 s would allow us to capture the full time course of the perceptual response. After returning to the start position, recordings finished when any perceptual after‐effects of the exposure had disappeared.

All trials were performed without vision. In experiment 1 this was achieved by eye closure. In experiment 2, trials were performed with eyes open in total darkness; this was to enable simultaneous eye movement recordings. Darkness was achieved by turning off room lights and blocking visible light sources. To aid this, a felt cloth draped over a frame formed a tent around the participant's head and the eye tracking equipment (all participants confirmed that this achieved complete darkness). Eye movement recordings in experiment 2 provide evidence of canal stimulation patterns and supplement limited existing data on the effect of head roll on nystagmus (see Discussion). Eye movement recordings were not done during experiment 1 because the limited space within the bore meant it was not possible to use our recording cameras across the full range of head pitch angles.

### Head orientations

Experiment one involved examination of seven target head pitch orientations: −80, −60, −40, −20, 0, +20 and +40 deg (Fig. 1 *C*; negative = extension; positive = flexion). Five were performed supine, whilst the two most extreme head extensions (−60 and −80 deg pitch) were performed prone (rationale below). Experiment 2 involved examination of five target head roll orientations: −40, −20, 0, +20 and +40 deg (negative = left ear to shoulder; positive = right ear to shoulder). These head roll orientations were performed supine with the head pitch orientation approximately 0 deg (Fig. 1 *D*). Supplementary trials were undertaken to identify the direction of the perception of rotation when head pitch was 180 deg and when head roll was 180 deg. Relative to neutral, 180 deg head pitch was achieved via feet first entry, prone body position, face down (Fig. 1 *E*, top), and 180 deg head roll was achieved via feet first entry, supine body position, face up (Fig. 1 *E*, bottom). Three participants undertook 180 deg head pitch trials and three different participants undertook 180 deg head roll trials. Whilst the main conditions used switch presses to log perception of rotation (see ‘Measurements’ section), only verbal descriptions of perception were recorded in these supplementary trials.

The reason for performing the −60 and −80 deg pitch conditions prone was because these head positions were difficult to comfortably maintain for several minutes when supine. In principle, rotating an individual about the long axis of the bore (*Z*_Lab_) so that they are prone instead of supine will have no effect on the *Z*‐orientated magnetic field relative to the head (it is equivalent to rotating the bore about a fixed head) and thus no effect on the field‐dependent vestibular input (Fig. 1 *F*). This notion was checked during pilot testing. Three participants who had all experienced perception of horizontal‐plane rotation upon entering the magnet supine with face pointing directly upward were also tested prone with their face pointing directly down (180 deg rotation about *Z*). As expected, in both orientations the perception of rotation was one of legs rotating to the right (from the participants’ perspective). For describing trends in the data, it is convenient to report the −60 and −80 deg head pitch data as if they were collected supine. To do this, we rotated the lab reference system used to describe perception of rotation by 180 deg about *Z*_Lab_ for these two conditions only (Fig. 1 *F*). Note that this is simply a transformation we have adopted for presentation purposes. Participants were never asked to think about reference systems, they simply reported perceptions in terms that made intuitive sense to them (e.g. legs rotating to their right in the horizontal plane).

We did not have a system to monitor three‐dimensional (3D) head orientation whilst inside the magnet. However, participants were aware we wanted their head to remain still, and to notify us if they felt that their head had actually moved. Only two of the 18 participants (in one to two trials each) reported they felt a possibility that their head may have moved a small amount whilst inside the magnet. Furthermore, in experiment 2, the 2D orientation of spectacle frames was monitored within the field of view of a camera positioned above the head (as part of the eye tracking procedure described later). Change in this orientation provided an approximation of any change in head roll orientation within the trial. When collapsed across conditions, mean ± SD of peak change in spectacle orientation (with respect to orientation at 0 T) was 0.0 ± 2.1 deg for signed change and 1.5 ± 1.6 deg for absolute change, with no significant difference between conditions in either case (*P* > 0.20).

### Measurements

Just prior to the start of each trial, photographs of the head were taken using digital cameras mounted on the wall and ceiling of the scanner room to measure head orientation and start position within the room.

Participants were asked to log their perception of horizontal‐plane rotation using an approach similar to that used in perceptual studies of physical rotation stimulation (e.g. Guedry & Lauver, 1961). Participants held an air‐operated switch in each hand. Each time they perceived themselves rotating through a prescribed angle, they would press a switch (Fig. 1 *B*). This angle was typically 45 deg, but we allowed the participants to use a different angle if they felt it would be easier. Using the time interval between switch presses, we calculated a perceived velocity of rotation at the time of each switch press (perceived velocity = angle/time interval). The direction of rotation was indicated by the switch that was pressed. One or two practice trials were performed at 0 deg head position (or at another head position if no rotation was perceived at 0 deg) prior to recorded trials. The primary focus of this study was on quantification of perception of horizontal‐plane rotation, although at the end of each trial, participants were also asked to describe any additional perceptions they had experienced, manipulating a small doll to assist their descriptions of motion.

For eye movement recording, an in‐house constructed Infrared camera (an adapted webcam) was mounted on a frame with a gooseneck arm, and positioned above one of the eyes (Fig. 1 *D*). Positioning was usually above the left eye for the neutral head orientation, and above the eye closer to the midline for non‐neutral head roll orientations. Infrared LEDs were used to illuminate the eye, and recordings were at 25–30 frames per second. Participants wore a pair of large spectacle frames (with lenses removed) which provided an initial head‐fixed reference frame for pupil tracking (subsequently converted to head coordinates). The video data were aligned with bed movement data using an audio tone generated by the analog acquisition system and captured in the video file.

An optical encoder (HEDS‐5701‐F00, Hewlett Packard, Palo Alto, CA, USA) coupled to one of the wheels of the bed was used to record the movement of the bed. Analog signals from the bed encoder and the hand‐held switches were captured onto a PC at 5 kHz through an analog‐to‐digital converter (USB‐6009, National Instruments, Austin, TX, USA). By combining the starting position of the head in room coordinates with the bed displacement data, we could localise the position of the head in room coordinates throughout the trial. The magnetic field experienced by the head was then determined using the magnetic field profile of the MRI room provided by Philips (Eindhoven, the Netherlands).

### Magnetic field exposure

Only the static magnetic field (*B*_0_) of the scanner was present in this study. At the isocentre it was 7 T and pointed in the −*Z*_Lab_ direction. Here we provide some analysis of the consequence of movement in and out of the magnet, and of changing head orientation, on the field exposure. When the bed is pushed into the scanner magnet bore, the strength of the static magnetic field experienced by the head gradually increases (Fig. 1 *B*). Thus, it is exposed to both a spatially varying (usually referred to as a magnetic field gradient) and a temporally varying magnetic field (d*B*_0_/d*t*; due to velocity through the gradient) only during movement of the bed. The point of maximum gradients, and hence d*B*_0_/d*t*, is near to the entrance to the bore. Three summary measures were used to characterise speed and d*B*_0_/d*t* experienced during bed movement. Collapsed across all head orientations and bed motion phases (in‐phase and out‐phase), the mean ± SD for these summary measures were 8 ± 3 cm s^−1^ (average bed speed), 0.8 ± 0.2 T s^–1^ (peak magnitude of d*B*_0_/d*t*) and 19 ± 4 s (duration of exposure to d*B*_0_/d*t* greater than 0.1 T s^–1^). None of these measures was significantly different between head orientation conditions (*P* > 0.05). Average bed speed was slightly (∼ 1 cm s^−1^) higher in the out‐phase than the in‐phase for the roll experiment only (*P* < 0.05).

Whilst the field is parallel to the long axis of the magnet bore at the isocentre (i.e. zero *B*_0_ *x* and *B*_0_ *y* components), off axis and away from the centre, the vestibular system will experience *B*_0_ *x* and *B*_0_ *y* components. A consequence of manipulating head angles in the current experiments was change in the radial position of the head within the magnet (head position varied vertically for pitch orientations and horizontally for roll orientations; Fig. 1 *C*, *D*). Taking into account the most extreme head positions, in the pitch experiment we measured that the vestibular system was never more than 12 cm from *Z*_Lab_ in both the *X*_Lab_ and the *Y*_Lab_ directions. In the head roll experiment, we measured that the vestibular system was never more than 6 and 20 cm from *Z*_Lab_ in the *X*_Lab_ and *Y*_Lab_ directions. respectively. Over a distance of ±60 cm from the isocentre along *Z*_Lab_, *B*_0_ *x* and *B*_0_ *y* remain essentially zero (< 0.1 T) even at the extreme head positions. Further from the centre (i.e. during travel into and out of the scanner), the *B*_0_ *x* and *B*_0_ *y* components become larger as the field begins to diverge. We estimate they would have magnitudes of no more than 0.41 T in the pitch experiment and 0.7 T in the roll experiment (given the *X*_Lab_ and *Y*_Lab_ distances mentioned above). These maximum *B*_0_ *x* and *B*_0_ *y* components would occur when the magnitude of *B*_0_ *z* is approximately 4.5 T.

### Analysis of switch press data

For analysis purposes, we split trials into an in‐phase (start of bed movement into the scanner until start of bed movement out of the scanner) and out‐phase (everything after the start of bed movement out of the scanner) (Fig. 1 *B*). Perception of horizontal‐plane rotation derived from switch presses was summarised in the same way as in our previous report of vertigo at the neutral head position (Mian *et al*. 2013). Onset of switch presses was the magnetic field experienced by the head at the instant of the initial switch press. Mean and peak velocity of perception were the mean and peak of the derived velocity time series (excluding the first switch press as velocity is indeterminate at this point). When participants did not report any perceived rotation (no switch presses), the duration and the mean and peak perception of rotation were assigned values of zero, and no value (a missing data point) was assigned to onset field. When participants only pressed the switch once (occurred for three trials in experiment 1, and three trials in experiment 2), they were reporting useful information on the presence and direction of perception of rotation, but the duration and magnitude was indeterminate. To avoid losing data, on these six trials we arbitrarily assigned values for duration of 5 s, and velocity magnitudes equal to the smallest of (1) 20% of the largest response of that participant at any tested head angle and (2) 20% of the largest response across all participants at the head angle condition of the trial.

### Analysis of qualitative descriptions of perception

Whilst the primary aspect of this study was assessment of the effect of head orientation on the perception of horizontal‐plane rotation logged by switch presses, qualitative descriptions of perception were also obtained. As a point of caution, it should be recognised that because these descriptions were provided at the end of each trial, they may be affected to some extent by recall uncertainty. Also, because the primary task of the participants was to log the perception of horizontal‐plane rotation, there may be some bias against formation of other perceptions. Nevertheless, the descriptions do provide significant additional information and hence have been summarised in this paper.

To summarise the descriptions, we coded them according to the presence or absence of different spatial components in the vertiginous response. Specifically, we noted whether or not the described vertigo involved perception of rotation about the vertical axis (*X*_Lab_) or the horizontal axes (*Y*_Lab_ or *Z*_Lab_), and whether it involved perception of non‐veridical translation. We ignored components suspected of being veridical. For example, participants occasionally reported a perception of travelling round a bend during bed movement. We suspected this involved a perception of rotation combined with a veridical perception of translation of the bed. Thus, in these cases we coded only for perception of rotation. We also coded trials according to whether or not vertiginous responses were described as nauseating or confusing.

### Analysis of eye movements

Eye movement analysis was as described in detail in prior reports (Mian *et al*. 2013; Glover *et al*. 2014) and a brief description is given here. 2D automated pupil tracking of the eye recordings was performed offline using in‐house written software which provided horizontal and vertical nystagmus slow‐phase velocities (velocity between nystagmus quick phases). Movements of the pupil in the video image were converted to ocular rotations using an assumption of 12 mm eyeball radius (Jansson, 1963) and reported as rotations about axes parallel to the head coordinate system (horizontal: ocular rotation about *Z*_Head_; vertical: ocular rotation about *Y*_Head_). Example time series from our recordings can be seen in prior reports (Mian *et al*. 2013; Glover *et al*. 2014). For group‐level reporting, we summarise the nystagmus eye movements by calculating the average slow‐phase velocities during the first 20 s at 7 T. Whilst torsional eye movements (ocular rotation about *X*_Head_) would have provided valuable additional information (see Discussion), our non‐specialist equipment was inadequate to obtain images showing the pattern/structure of the iris and so torsion could not be inspected or quantified.

### Missing and excluded trials

A complete data set for experiment 1 (head pitch experiment) would comprise 84 trials (seven conditions × 12 participants) and for experiment 2 (head roll experiment) would comprise 55 trials (five conditions × 11 participants). However, some participants did not attempt every condition. In experiment 1, 10 of 12 participants attempted all seven conditions. One participant attempted only six conditions (due to nauseous sensations during the sixth trial) and one attempted only five conditions (due to time constraints). In experiment 2 (head roll manipulations), 10 of 11 participants attempted all conditions. One participant withdrew after three trials (owing to development of nausea).

All attempted trials were included in the qualitative analysis of perceptions. However, some attempted trials were excluded from numerical analysis of switch presses. In experiment 1, three trials were excluded due to early termination (two at −60 deg and one at −80 deg) and four trials were excluded because, despite completing the trial, the participants found the vertigo too confusing to confidently report the perception (two at −60 deg and two at −80 deg). Two additional trials were excluded during other conditions (one at +40 deg and one at −20 deg) due to the participants expressing uncertainty that they had executed switch presses correctly. In experiment 2, no attempted trials were excluded. The net result of non‐attempted trials and excluded trials is that 72 (experiment 1) and 53 (experiment 2) trials were included in numerical analysis of the perception of horizontal‐plane rotation. The distribution of included trials across head orientations is provided in Table 1 for the various measures used in the study.

**Table 1:** Number of participants included in qualitative descriptions and switch press measures

	Target head angle (degrees)						
	−80	−60	−40	−20	0	20	40
Pitch experiment							
Qualitative descriptions	11	12	12	11	12	11	12
Switch‐press measures							
DI, MV, PV, D	8	8	12	10	12	11	11
OF, in‐phase	7	3	10	9	10	2	4
OF, out‐phase	5	3	7	7	8	3	2
Roll experiment							
Qualitative descriptions	N/A	N/A	10	11	11	11	10
Switch‐press measures							
DI, MV, PV, D	N/A	N/A	10	11	11	11	10
OF, in‐phase	N/A	N/A	8	8	10	8	4
OF, out‐phase	N/A	N/A	5	7	8	8	6

All attempted trials contributed to qualitative description data (Tables 2 and 3), although not every participant attempted every condition (see main text). For quantitative data derived from switch presses (Figs 3 and 4; Table 4), some attempted trials were excluded (see main text). The numbers of participants for direction‐specific incidence (DI; Fig. 3), mean velocity (MV; Fig. 4), peak velocity (PV; Fig. 4) and duration (D; Fig. 4) measures are the same, and represent the number of participants remaining after exclusion of some attempted trials. Unlike the aforementioned measures, onset field (OF; Fig. 4) is dependent on the presence of at least one switch press. Hence, for this measure fewer participants contribute data points, and different numbers contribute to the in‐phase and out‐phase.

Eye movement data for three of the 11 participants in experiment 2 were unavailable (one due to early termination of the experiment, one due to no attempted recordings and one due to poor video quality). However, eye movements were recorded in two additional participants who did not provide perceptual reports (but met the same study inclusion criteria) and are included here. Thus, the resultant sample size for the eye movement data is 10. In many of the participants, eye movements were not recorded for the ± 20 deg conditions. Thus, we have limited analysis to the 0 and ± 40 deg conditions.

### Statistics

We used linear mixed models to test for effects of head orientation and trial phase on summary measures of perception of horizontal‐plane rotation (SPSS, v20). Target head orientation and trial phase (in‐phase, out‐phase) were fixed factor predictors, with participants as random factors. Mixed models were used due to their advantage in dealing with missing data. Unlike general linear models (e.g. ANOVA), which discard entire cases (subjects) when data from single trials are missing, mixed models make use of all available data. Perception of rotation during the out‐phase is in the opposite direction to the in‐phase and commences at a low field rather than a high field (Fig. 1 *B*). It is not of interest to test these obvious differences statistically. Furthermore, when in‐phase and unadjusted out‐phase data are included in the same statistical model they could mask main effects of head orientation and could confound interaction effects. Thus, to incorporate the in‐phase and out‐phase in the same statistical model, we inverted the out‐phase perception velocity (multiplied by −1) and expressed onset field as the *magnitude* of change in field from the start of bed movement (e.g. an onset field of 1 T during the out‐phase was expressed as a 6 T change from start of bed movement: magnitude of 1 T minus 7 T). Linear mixed models were also used for statistical analysis of the effect of head roll on nystagmus slow phase velocity. An alpha level of 0.05 was used for judging statistical significance.

## Results

### Measured head orientations

Group mean ± SD measured head pitch orientations for the seven conditions in experiment 1 were −78 ± 9, −56 ± 5, −40 ± 5, −23 ± 8, −1 ± 8, 23 ± 5 and 42 ± 4 deg. Measured head roll orientations for the five conditions in experiment 2 were −34 ± 7, −18 ± 4, 0 ± 2, 16 ± 4 and 33 ± 5 deg. Thus, whilst attained head pitch orientations were generally close to target head orientations, head roll orientations slightly undershot their targets. Nevertheless, we will refer to the conditions according to target head orientation for the remainder of the paper.

### Qualitative description of perceptions

On a number of trials participants described the vertigo as confusing or nauseating. On these trials, participants sometimes stopped paying attention, and opened their eyes, as soon as they were withdrawn from the magnetic field. Whilst such trials were entirely excluded from the analysis of switch press data (‘Missing and excluded trials’ in Methods), they are included in this qualitative summary. However, to avoid bias between in‐phase and out‐phase due to unbalanced data, we restrict this qualitative summary to the descriptions given for the in‐phase only. The observed incidence of various perceptions described below as well as incidence of early withdrawals are given in Table 2 (head pitch experiment) and Table 3 (head roll experiment).

**Table 2:** Incidence (%) of perceptions during the in‐phase and incidence (%) of early withdrawals in the head pitch experiment

	Target head angle (degrees)						
	−80	−60	−40	−20	0	+20	+40
Any vertigo/dizziness	91	100	92	91	83	18	50
Rotation about *X*_Lab_	64	25	92	91	83	18	33
Rotation about *Y*_Lab_	0	17	0	0	0	0	25
Rotation about *Z*_Lab_	9	50	17	9	0	0	8
Translation	9	17	0	9	0	0	0
Confusing	27	33	0	9	0	0	8
Nauseating	45	50	0	0	0	0	0
Early withdrawal	9	17	0	0	0	0	0

Incidence (%) equals 100 × (number of participants with perception) / (number of participants attempting the head angle condition). The number of participants attempting each head angle is given in the qualitative descriptions row of Table 1 (pitch experiment).

**Table 3:** Incidence (%) of perceptions during the in‐phase and incidence (%) of early withdrawals in the head roll experiment

	Target head angle (degrees)				
	−40	−20	0	+20	+40
Any vertigo/dizziness	80	73	91	73	40
Rotation about *X*_Lab_	80	73	91	73	40
Rotation about *Y*_Lab_	10	9	0	9	0
Rotation about *Z*_Lab_	0	0	18	0	0
Translation	0	0	0	0	0
Confusing	0	0	0	0	0
Nauseating	0	9	0	18	0
Early withdrawal	0	0	0	0	0

Incidence calculated as in Table 2. The number of participants attempting each head angle is given in the qualitative descriptions row of Table 1 (roll experiment).

Dizziness or vertigo of any form was reported by the majority of participants at neutral and head extension (negative head pitch) conditions, but was less common during head flexion (positive head pitch) conditions. It was also reported by the majority of participants across the head roll conditions, with the exception of +40 deg head roll. Within the range −40 to +40 deg of both head pitch and roll, the qualitative characteristics of the vertiginous perceptions were generally the same as we previously described in detail for the neutral head position (Mian *et al*. 2013), albeit with modulation of intensity, duration and direction. Specifically, the most common perception was one of horizontal‐plane rotation (rotation about *X*_Lab_). This can be described as a dynamic perception of the bed being rotated about a vertical axis, with a duration that sometimes persisted for over 1 min. We did not routinely ask participants to attend to the perceived centre of rotation, but when sometimes asked after a trial, estimates varied between the centre of the head and the navel. At these head orientations, self‐motion perceptions other than horizontal‐plane rotation were uncommon. Occasionally participants described perceptions of rotation about *Y*_Lab_ (i.e. leg or head being raised) or *Z*_Lab_ (i.e. rotation about long axis of body) or non‐veridical translation. However, these perceptions were relatively short (a few seconds), and in the case of rotation sometimes static rather than dynamic. Participants rarely described the vertiginous perceptions within the ± 40 deg head pitch or roll range as confusing or nauseating.

It was at the two most extreme head extension conditions that differences (from neutral) in the qualitative characteristics of vertigo were most notable. At −60 deg head pitch, a perception of rotation about *Z*_Lab_ was fairly common. At both −60 and −80 deg head pitch, it was common for participants to describe the vertigo as being complex or confusing, sometimes being unable to provide a clear description. A complex perception is in line with some of our own experiences at these head orientations during pilot experiments. It was felt that the complexity involved a strong feeling of simultaneous rotation about multiple axes, with perception of rotation about horizontal axes being more persistent than at other head orientations. Although the naive volunteers did not spontaneously offer such a description, under suggestion they agreed that this was a possible interpretation of their confusing perceptions. Many participants described the experience at these head positions as nauseating with a few requesting early termination of the trial.

### Quantitative analyses of perceived horizontal‐plane rotation

#### Effect of head pitch

**Figure 2:**
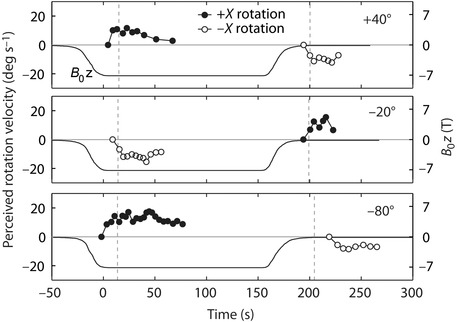
**Examples of perceived horizontal‐plane rotation** Each panel is a single trial example from the same participant at different target head pitch orientations (+40, −20 and −80 deg). Perceived rotation velocities (circles; left axis) derived from time intervals between switch presses are plotted against time. Each circle denotes the instant of a switch press. The first circle in each series is arbitrarily plotted at 0 deg s^–1^ because magnitude is indeterminate at this instant. White circles are used for −*X*_Lab_ rotation direction and black circles for +*X*_Lab_ rotation direction. The solid curve represents the instantaneous *B*_0_ *z* experienced by the head (right axis). The vertical dotted lines are when the bed stops moving during the in‐phase and the out‐phase. For presentation purposes, data have been horizontally aligned such that the instant the head reaches 7 T during the in‐phase is at 0 s.

Figure 2 shows example data of the perceived rotation velocity time‐series at three head pitch conditions from a representative participant. At −20 deg head pitch, the perceived rotation was in the −*X*_Lab_ direction during the in‐phase, and in the +*X*_Lab_ direction during the out‐phase, as previously reported for 0 deg head pitch (Mian *et al*. 2013). However, for +40 and −80 deg head pitch angles the perceived rotation was in the opposite direction (+*X*_Lab_ in‐phase; −*X*_Lab_ out‐phase).

**Figure 3:**
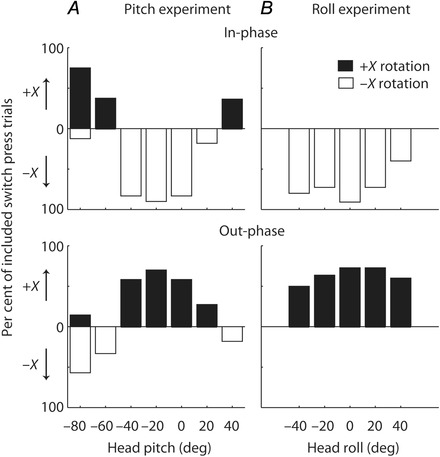
**Direction‐specific incidence of perceived horizontal‐plane rotation** Each plot shows incidence of reporting of +*X*_Lab_ direction of rotation (upward black bars) and −*X*_Lab_ direction rotation (downward white bars) during the in‐phase (top panels) and out‐phase (bottom panels) for the pitch experiment (*A*, left column) and roll experiment (*B*, right column). Incidence represents percentage of the included switch press trials (DI rows in Table 1).

Figure 3 *A* shows the incidence of each direction of perceived horizontal‐plane rotation during the in‐phase and out‐phase at the group level. The trend that emerges is consistent with the example data of Fig. 2. Namely, the magnetic field causes −*X*_Lab_ rotation at −40 to +20 deg head pitch angles and, when the perception is present, there is generally a reversal of the direction of rotation (+*X*_Lab_) for the more extreme head extension and flexion angles. Furthermore, the group summary confirms that reversal of the perception of rotation during the out‐phase is generalised across participants and head orientations. In the trials at 180 deg head pitch performed by a subset of participants (*n* = 3), two reported perception of horizontal‐plane rotation in the −*X*_Lab_ direction (legs to their left for this condition; cf., Fig. 1 *E*, top) during the in‐phase. The other participant, who had not experienced perception of horizontal‐plane rotation at 0 deg head pitch, also did not experience perception of rotation at 180 deg head pitch.

**Figure 4:**
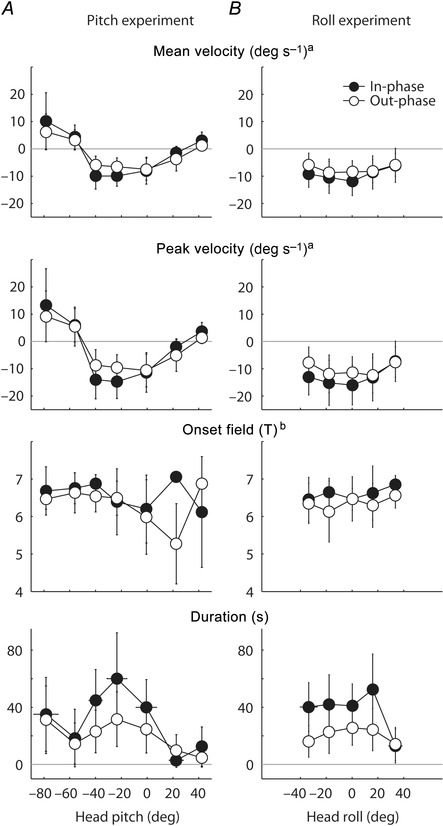
**Summary measures of perceived horizontal‐plane rotation** Each plot shows mean (for each target head orientation) of measures derived from switch presses during the in‐phase (black circles) and out‐phase (white circles) plotted against mean measured head orientation. Vertical error bars denote 95% confidence intervals. Horizontal error bars (drawn only for the in‐phase symbols in the bottom panels) denote standard deviation of measured head orientations. For this figure, out‐phase rotation velocities and onset field have undergone transformation to aid visual inspection of the phase × condition interaction: ^a^out‐phase rotation velocities have been multiplied by −1; ^b^out‐phase onset field has been expressed as magnitude of change in *B*_0_ *z* relative to in‐phase *B*_0_ *z* (7 T). Thus, a 1 T out‐phase onset field is expressed as |1 T − 7 T| = 6 T. The number of participants contributing to each measure is shown in Table 1.

Figure 4 *A* plots summary data for perceived rotation velocity, duration and onset field, with the results of associated statistical tests in Table 4. For this figure and statistical tests, out‐phase rotation velocity and onset field data have been transformed as described in the *Statistics* section of Methods. Statistical tests revealed a significant main effect of head pitch on the mean and peak perceived rotational velocity and the duration of perception (*P* < 0.01). There was a main effect of phase on duration of perception (*P* < 0.01) with duration being longer during the in‐phase, but the effect was limited to −40 to 0 deg pitch angles producing a phase × pitch interaction (*P* < 0.01). There was a significant interaction effect for onset field (*P* = 0.042), seemingly due to phase differences at the +20 deg head pitch (Fig. 4 *A*). This effect is unlikely to be robust because the number of participants with onset field data at this head angle is low (Table 1; OF measure).

**Table 4:** Statistical tests of perceived horizontal‐plane rotation

	Mean velocity		Peak velocity		Onset field		Duration	
	*F*	*P*	*F*	*P*	*F*	*P*	*F*	*P*
Pitch experiment								
Trial phase	0.01	0.93	0.02	0.89	0.97	0.33	2.4	0.13
	[1, 51]		[1, 48]		[1, 37]		[1, 43]	
Head angle	14.6	< 0.001	14.0	< 0.001	1.62	0.16	6.7	< 0.001
	[6, 95]		[6, 93]		[6, 48]		[6, 99]	
Phase × Angle	0.84	0.54	0.63	0.71	2.39	0.042	1.2	0.32
	[6, 94]		[6, 92]		[6, 49]		[6, 96]	
Roll experiment								
Trial phase	0.62	0.43	0.67	0.42	1.26	0.27	6.13	0.017
	[1, 49]		[1, 48]		[1, 30]		[1, 44]	
Head angle	1.84	0.13	2.31	0.065	0.41	0.80	5.75	< 0.001
	[4, 80]		[1, 79]		[4, 44]		[4, 78]	
Phase × Angle	0.50	0.74	0.46	0.77	0.65	0.63	2.17	0.080
	[4, 79]		[4, 79]		[4, 45]		[4, 77]	

Table shows main effects of linear mixed model analysis. Numerator and denominator degrees of freedom are in square brackets. Denominator degrees of freedom are given to the nearest whole number.

#### Effect of head roll

The direction‐specific incidence of perceived rotation during the in‐phase and out‐phase is plotted in Fig. 3 *B* showing that the direction of the perception of horizontal‐plane rotation was −*X*_Lab_ during the in‐phase and +*X*_Lab_ during the out‐phase across all head angles from −40 to +40 deg. In the trials at 180 deg head roll performed by a subset of participants (*n* = 3), all reported perception of horizontal‐plane rotation in the +*X*_Lab_ direction during the in‐phase (cf. Fig. 1 *E*, bottom).

Figure 4 *B* plots summary data for perceived rotation velocity, duration and onset field, with the results of associated statistical tests in Table 4. Again, the out‐phase rotation velocity and onset field data have been transformed as described in Methods. There were no significant main effects for mean velocity, peak velocity or onset field at an alpha level of 0.05. However, the main effect of head roll on peak velocity was close to the alpha level (*P* = 0.065). Duration of perception was significantly shorter during the out‐phase (*P* = 0.017). There was a significant main effect of head roll on duration (*P* < 0.001) and with phase × roll interaction close to the alpha level (*P* = 0.080). The head angle effect on duration appears to be primarily driven by the relatively short duration of perception during the in‐phase at +40 deg head roll. This can be attributed to the relatively low incidence of in‐phase perception (i.e. high incidence of recording 0 s duration responses) at this head angle (Fig. 3 *B*).

#### Aberrant responses

Two additional observations from the switch press data are mentioned here. Due to their low incidence, we cannot say whether they are related to head orientation. However, they may be considered generally relevant to the proposed mechanism of vestibular stimulation and will be considered in the Discussion.

**Figure 5:**
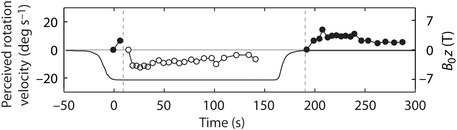
**Within trial phase reversal of perceived horizontal‐plane rotation** Meaning of symbols and lines are as in Fig. 2. This example illustrates the rare occurrence of a change in direction of perceived rotation within the in‐phase. The initial two switch presses denoting +*X*_Lab_ rotation during the in‐phase in this trial were ignored when calculating summary statistics.

(1) Whilst perception of horizontal‐plane rotation generally consisted of one direction of rotation during the in‐phase and the reverse direction during the out‐phase (Fig. 2), a small number of trials (6 of 125 trials across the two experiments; two at −20 deg pitch, two at −40 deg pitch, one at −40 deg roll, one at +20 deg roll) exhibited a brief initial period (1–2 switch presses) of apparent rotation in one direction which then reversed to a longer lasting perception *within* the same movement phase (Fig. 5). When this occurred we did not include the initial switch presses in the calculation of the summary measures described earlier.

(2) There were a small number of trials (5 of 125) in which perception of rotation during the out‐phase was present in the absence of in‐phase perception of rotation.

### Eye movements

**Figure 6:**
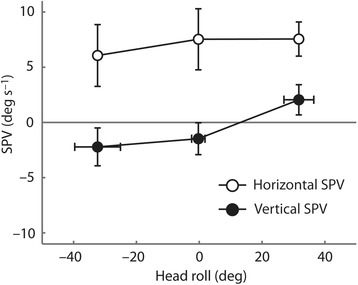
**Effect of head roll on nystagmus slow‐phase velocity** Group average horizontal (ocular rotation about *Z*_Head_) and vertical (ocular rotation about *Y*_Head_) slow‐phase velocity (SPV; averaged during first 20 s at 7 T) plotted against average head roll angle for the neutral position and the two extreme head roll angles (*n* = 10). Vertical error bars denote 95% confidence intervals. Horizontal error bars (drawn only once per condition) denote standard deviation of measured head roll.

Figure 6 shows average horizontal and vertical nystagmus slow phase velocities (SPVs) averaged over the first 20 s at 7 T. Horizontal SPV was leftward (+*Z*_Head_) at each head orientation with no significant difference across conditions (*F*_2,18_ = 1.57, *P* = 0.23). Vertical SPV was significantly different across conditions (*F*_2,18_ = 12.2, *P* < 0.01). Notably, the average SPV was downward (+*Y*_Head_) for the right‐ear‐to‐shoulder (positive roll) orientation and upward (–*Y*_Head_) for the left‐ear‐to‐shoulder (negative roll) orientation.

## Discussion

This study has shown that static magnetic fields produce non‐veridical perceptions of self‐motion (vertigo) that are significantly affected by static head orientation. In this discussion, we consider the observations mainly within the context of the Lorentz force hypothesis of vestibular stimulation by magnetic fields described in the Introduction. Dependence of vertigo on the orientation of the head relative to the magnetic field does not, in itself, rule out other suggested mechanisms. However, under our experimental conditions of slow travel into the magnet bore, and static uniform fields for the majority of the exposure period, the alternative mechanisms are considered less likely (refer to Introduction and Mian *et al*. 2013).

We begin this discussion by considering whether the periodicity of the relationships between head orientation and perceived horizontal‐plane rotation are compatible with what may be expected on theoretical grounds (first section). An understanding of these relationships will not only support our general understanding of magnetically induced vertigo, but also inform a subsequent discussion (second section) of whether magnetically induced vertigo and nystagmus are driven by the same vestibular signal. The remaining sections of the discussion consider other observations that emerged from the experiments.

### Relationship between head orientation and perception of horizontal‐plane rotation

**Figure 7:**
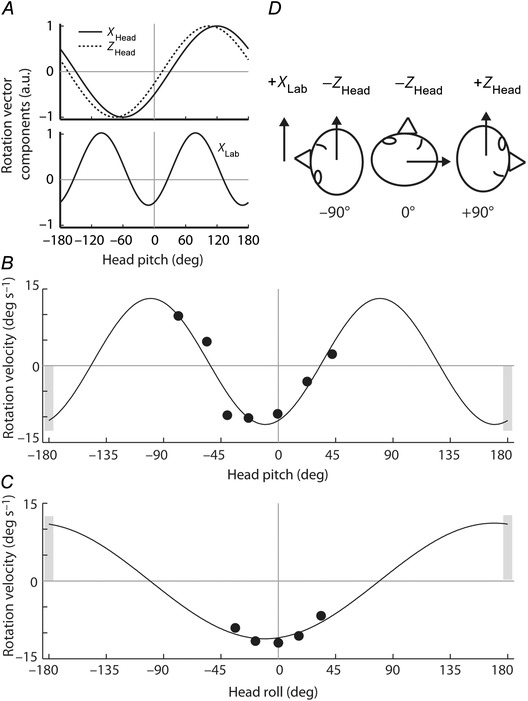
**Sinusoidal variation between head angle and magnetically induced vestibular signal and vertigo** *A*, the top panel shows sinusoidal relationships (of spatial frequency = one cycle per 360 deg pitch) between head pitch and *X*_Head_ and *Z*_Head_ components of a hypothetical magnetically induced rotational vestibular signal. The transition from a −*Z*_Head_ to +*Z*_Head_ rotation signal as the head moves from extension to flexion is broadly based on observations of horizontal (left–right) nystagmus by Roberts *et al*. (2011), who used the same magnetic field polarity as the current study. The relative phase and magnitude of the *X*_Head_ rotation component is arbitrary and for illustrative purposes. The bottom panel shows that projection of these rotation signals to *X*_Lab_ leads to a sinusoid with a spatial frequency of two cycles per 360 deg: *X*_Lab_ = (*X*_Head_ × cos θ) + (*Z*_Head_ × sin θ), where θ is head pitch angle. The offset, amplitude and phase of *X*_Lab_ rotation signal would vary depending on the relative amplitude and phase of *X*_Head_ and *Z*_Head_, but the spatial frequency will always be 2. *B*, sinusoid (with fixed frequency = 2) fitted to the relationship between head pitch and perceived rotation velocity data. *C*, sinusoid (with fixed spatial frequency = one cycle per 360 deg roll, and fixed offset = zero), fitted to perceived rotation velocity data plotted against head roll. The data points in *B* and *C* represent the overall average of the in‐phase and transformed out‐phase mean and peak velocity data (i.e. top two rows in Fig. 4). Fitted curves have been extrapolated to ± 180 deg head orientations. The shaded bars at ± 180 deg depict the polarity (but not magnitude) of perception of rotation at these orientations identified during the supplementary trials. *D*, cartoon showing expected direction (not magnitude) of the *Z*_Head_ component of magnetically induced rotational vestibular signal based on the relationship between head pitch and horizontal nystagmus (see *Z*_Head_ in *A*). For −90 and +90 deg head pitch, the *Z*_Head_ component has opposite polarities in head coordinates. Because of this, they point in same direction in space as +*X*_Lab_.

Roberts *et al*. (2011) put forward a simple geometric model of lateral canal transcupular pressures induced by static magnetic field‐induced Lorentz forces. Antunes *et al*. (2012) subsequently described a more complex simulation of lateral canal pressures which incorporated fluid dynamics. Both models suggest that the relationship between head pitch and lateral canal pressures is sinusoidal with a spatial frequency of 1 (one cycle per 360 deg of head pitch), and one would expect this frequency to generalise to other canals. Output from individual canals can be assumed to represent neural equivalents of rotation vectors perpendicular to the plane of the canals. The components of these vectors in head coordinates can be summed to produce a single net rotation vector. Because the sum of *N* non‐cancelling sinusoids of frequency *F* will always have frequency *F* (Weisstein, date unknown), it follows that the relationship between head pitch and the components of the net rotation vector *in head coordinates* (*X*_Head_, *Y*_Head_, *Z*_Head_) will also have a spatial frequency of 1 (Fig. 7 *A*, top). However, our representation of the perception of horizontal‐plane rotation is *in laboratory coordinates* (rotation about *X*_Lab_) and therefore represents a signal of rotation transformed from head to laboratory coordinates. This transformation causes the relationship between head pitch and the *X*_Lab_ rotation component to be a sinusoid with a spatial frequency of two cycles per 360 deg pitch (Fig. 7 *A*, bottom).

Using the MATLAB function *nonlinearmodel.fit*, we fit a sinusoid to our group mean data on perceived horizontal‐plane rotation velocity (*V*) as a function of static head pitch angle (θ): *V* = *O* + *A* sin (ω θ + ϕ). Offset (*O*), amplitude (*A*) and phase (ϕ) were fitted parameters and spatial frequency (ω) was fixed at two cycles per 360 deg head pitch. Offset was a fitted parameter because although in head coordinates we would expect a zero offset for the rotation signal, this is not necessarily the case after transformation to lab coordinates (cf. Fig. 7 *A*, bottom). The fit to the data is represented in Fig. 7 *B* showing compatibility with the reasoning laid out above. Ideally, a wider range of perceived rotation velocities is desirable for increased confidence in the fit. However, an additional test was performed to check that the spatial frequency of the relationship was indeed 2 instead of 1. If true, the direction of the perception of rotation in lab coordinates should be the same at ± 180 deg pitch as at 0 deg pitch, which was the case (shaded segments in Fig. 7 *B*).

Unlike pitch, when the head is rotated in roll there is no change in the inclination of the head coordinate system with respect to the horizontal plane and so *X*_Lab_ remains approximately equal to *X*_Head_. Therefore, any relationship between head roll and perception of horizontal‐plane rotation can be expected to be sinusoidal with a zero offset and a spatial frequency of one cycle per 360 deg roll. Although the statistical test of the effect of head roll on perceived rotation velocity did not demonstrate statistical significance at *P* < 0.05 (probably due to a limited range of head orientations, which was constrained by bore diameter), a sinusoid with spatial frequency 1 and zero offset is compatible with the data (Fig. 7 *C*). This model is supported by the observation that ± 180 deg head roll produces a reversed direction of perceived rotation in lab coordinates (shaded bars in Fig. 7 *C*).

### Are vertigo and nystagmus induced by the same vestibular signal?

At the neutral head position (lab and head coordinate systems aligned), healthy participants tend to exhibit robust horizontal (i.e. left–right) nystagmus (Fig. 6, and previously: Roberts *et al*. 2011; Glover *et al*. 2014) without obvious torsional nystagmus (Ward *et al*. 2014). The former suggests the presence of a vestibular signal of rotation about the *Z*_Head_ axis whilst the latter suggests an absence of a vestibular signal of rotation about *X*_Head_. In contrast, at this same head orientation participants tend to experience clear perception of rotation about *X*_Lab_/*X*_Head_ whilst perception of rotation about *Z*_Lab_/*Z*_Head_ is absent or relatively short‐lasting. This striking discrepancy in the spatial properties of nystagmus and vertigo represents a challenge if they are to be interpreted as emerging from the same vestibular input.

Toward reconciliation of this discrepancy, it should be recognised that stimulation of only the semicircular canals (as per the Lorentz force hypothesis) can produce an unnatural multisensory conflict that the brain must resolve. This occurs when one set of organs (semicircular canals) signals a change in body orientation with respect to gravity while other sets (otoliths, cutaneous receptors, somatic graviceptors) report no change. It has been suggested that perceptual processes combine such multisensory cues according to a Bayesian framework (Angelaki *et al*. 2009). In this model, the perceptual outcome depends on the relative reliability of the individual cues and the distribution of prior expectation. An absence of perception of vertical‐plane rotation, even when signalled by semicircular canals, could then result from the greater number of receptors signalling no change in orientation with respect to gravity and a prior favouring no rotation inside an MRI scanner. In contrast, perception of horizontal‐plane rotation could survive when signalled by semicircular canals because it would be resisted only by the prior and not by other sensory signals (Mian *et al*. 2013, 2015). The vestibulo‐ocular reflex, on the other hand, employs simpler neural circuitry and therefore probably is less influenced by multisensory cue integration processes. Additionally, as we have suggested before (Mian *et al*. 2015), the considerably smaller gain of torsional compared to horizontal nystagmus (Bockisch *et al*. 2005) may mean that neither nystagmus nor perception faithfully represents the vector of the underlying magnetically induced vestibular signal.

A related issue is whether there is evidence of suitable canal stimulation patterns. Whilst evidence of lateral canal stimulation required for the Lorentz force mechanism is provided by the presence of horizontal nystagmus (Roberts *et al*. 2011), evidence of vertical canal stimulation is necessary to account for simultaneous perception of rotation about *X*_Lab_ when the head is supine. This is because a perception of body rotation in the earth‐horizontal plane arising from the lateral canals would be in the opposite direction to that observed (Mian *et al*. 2015). In this regard, although there is minimal vertical eye movement in the neutral head position, change in vertical eye movements are induced when the head is re‐positioned in roll, as reported previously using two participants (Roberts *et al*. 2011) and now confirmed with a larger sample in the current study (Fig. 6). Furthermore, magnetically induced vertical eye movements at the neutral position have recently been observed in patients with unilateral hypofunction in a way that suggests the presence of anterior canal signals that cancel bilaterally in terms of their contribution to vertical eye movements in healthy participants (Ward *et al*. 2014). Interestingly, the roll component of the inferred anterior canal signals would sum bilaterally in way that is compatible with the observed direction of perception (Mian *et al*. 2015).

Given the equivocacy inherent in trying to reconcile magnetically induced nystagmus and perceptions that are largely orthogonal when the head is supine, the discussion of whether these effects are driven by the same signal can perhaps be best advanced by considering head positions at which the measured perception of rotation (i.e. about *X*_Lab_) and the robust horizontal nystagmus (ocular rotation about *Z*_Head_) are both in the same plane. This would happen when the head is ± 90 deg from supine (Fig. 7 *D*). Evidence supportive of a common signal would be provided if the polarity of perception and nystagmus are compatible. Whilst these head positions have not been studied (it was not possible to achieve a forward flexion to an upright head position within the confines of the bore) our new understanding of the relationship between head pitch and perception, determined in the previous section, enables us to effectively extrapolate to these positions. This can be combined with an understanding of the effect of head pitch on eye movements (Roberts *et al*. 2011). When the head is increasingly extended from the neutral position, the velocity of the slow phase of nystagmus increases in the leftward (+*Z*_Head_) direction. Conversely, a rightward slow‐phase velocity (–*Z*_Head_) increases with head flexion (Roberts *et al*. 2011). Because the slow‐phase of nystagmus is compensatory, this implies an increasing −*Z*_Head_ vestibular rotation signal with head extension and an increasing +*Z*_Head_ vestibular rotation signal with head flexion. Assuming these polarities are maintained at ±90 deg head pitch (in line with expectation for one cycle per 360 deg, Fig. 7 *A*, top), for both angles this is equivalent to a vestibular rotation signal that points upwards in lab coordinates (+*X*_Lab_) (Fig. 7 *D*). This is indeed compatible with the +*X*_Lab_ direction of perceived body rotation predicted by extrapolation of the sinusoid fitted to the perception data at ±90 deg pitch (Fig. 7 *B*).

### Nauseating and confusing perceptions

The most nauseating trials were the −60 and −80 deg head pitch trials. Perceptions at these orientations were often described as complex or confusing. Although not universal, some of our own experiences during pilot testing were that these complex perceptions involved simultaneous perception of rotation in multiple planes. We can only speculate as to why this was most common at these head angles. As noted in the previous section, we suspect the perception of horizontal‐plane rotation is generally dominant not because the induced vestibular signal indicates only rotation in this plane, but because this component does not conflict with veridical signals indicating the body is stationary in the vertical plane. One possibility is that at certain head orientations, components of the magnetically induced vestibular rotation signals about horizontal axes may become large enough to overcome these veridical signals leading to confusing, multi‐planar perceptions of motion.

Note that of the conditions with switch press data, −60 and −80 deg head pitch were the only ones performed prone. However, it seems unlikely that ventral instead of dorsal body surface contact with the bed per se is responsible for the greater incidence of complex, nauseating perceptions in these conditions. This is because none of the other conditions performed prone (180 deg pitch trials, Fig. 1 *E*; neutral head position prone trials in pilot testing, Fig. 1 *F*) induced such responses. Note also that a head extension position itself can sometimes provoke vertigo (Brandt & Daroff, 1980). However, because participants did not report vertigo in the period prior to entry into the magnet, it seems unlikely that head extension itself is responsible for the more severe vertigo in the head extended conditions in the current study.

Interaction between dynamic canal signals (induced by the magnetic field) and tonic head and neck sensory signals, or postural task requirements could be factors. In examining the effect of orientation of the magnetic field relative to head, the purest mode of investigation would be to rotate the magnetic field about the head, which is not possible with an MRI scanner. Thus, in this study, as in other related studies (Roberts *et al*. 2011; Houpt *et al*. 2013), the head is rotated relative to a fixed magnetic field. This involves changes in the direction of gravity relative to the head, and thus altered tonic stimulation of the otoliths by gravity. Furthermore, even though a head/neck support frame was used in the current study, it is highly probable that there was variation in postural muscle activity between conditions.

### Aberrant perceptions of horizontal‐plane rotation

We noted a few cases of departure from the usual pattern of perception of horizontal‐plane rotation. Although not related to the main question of the effect of head orientation, these observations may be considered relevant to the more general issue of the mechanism of magnetic vestibular stimulation and are discussed here.

On a small number of trials (6 of 125), a reversal of the perception of rotation was sometimes reported within a trial phase (Fig. 5). When this occurred, it was generally during, or just after, the head was travelling through the magnetic field gradient. If the Lorentz force hypothesis is true, a constant magnetic field (as at the isocentre) is analogous to a constant angular acceleration and a gradually changing magnetic field (as during slow entry/exit into the magnet) is analogous to a ramp angular acceleration. Clarke & Stewart (1968) studied perception of rotation during both constant and ramp angular accelerations about a vertical axis and report that during ramp angular accelerations, patterns of perception were sometimes complex and bizarre. For example, ‘I feel that I am rotating in both directions at the same time’ (Clark & Stewart, 1968, p. 335). Therefore, occasional instances of complexity in the dynamics of perception of horizontal‐plane rotation noted in the current study, involving changing of direction or uncertainty in direction of perception, are not necessarily incompatible with the Lorentz force hypothesis. An alternative explanation of these observations could be that an additional stimulus, such as current induced by temporally changing magnetic field stimulating the vestibular nerve (Glover *et al*. 2007), has a transient effect over and above the primary stimulus.

Perception of rotation was sometimes present after withdrawal from the field even in the absence of perception of rotation during exposure to the field. This might be unexpected as we favour interpretation of the perception upon withdrawal as an after‐effect of perceptual adaptation to the field (Mian *et al*. 2013). Thus, it might be expected that perception during stimulation is a prerequisite for perception after stimulation. However, Clarke & Stewart (1968) also reported that perception of rotation during angular acceleration stimulation was not a prerequisite for perception of an after‐effect. Specifically they noted that on 19 out of 350 trials (5.4%) across 10 participants and various acceleration profiles, post‐rotation perception was reported in spite of absence of per‐rotation perception. In our experiments, such behaviour was present in 4% of the trials included in our quantitative analysis of the perception of horizontal‐plane rotation (5 of 125).

### Conclusions

We have found that there is a significant effect of static head orientation on vertiginous perceptions in static magnetic fields. The shape of the relationship between head orientation and perception of horizontal‐plane rotation is compatible with that expected by the Lorentz force hypothesis of semicircular canal stimulation by magnetic fields. The effects stem from simultaneous activation of multiple canals together with transformation of the net craniocentric vestibular signal to the earth horizontal plane. Furthermore, the change in the direction of perception as a function of head pitch is compatible with the direction of previously studied magnetically induced nystagmus. Observations of practical value were that vertigo becomes more complex and nauseating as head extension is increased and is weakest if the head is flexed with Reid's plane approximately 20 deg forward of vertical.

## Additional information

### Competing interests

None.

### Author contributions

Conception and design: OSM, YL, AA, PG BLD; data collection: OSM, YL, AA; data analysis: OSM, YL, AA; data interpretation: OSM, YL, AA, PG BLD; initial manuscript preparation: OSM, BLD; manuscript editing: OSM, YL, AA, PG BLD. Experiments were carried out at the University of Nottingham.

### Funding

The work was supported through funding from the UK Engineering and Physical Sciences Research Council (grant numbers EP/G062692/1 and EP/G061653/1).
